# Association Between Serum Vitamin D Levels and Retinal Vascular Occlusion: A Systematic Review and Meta-Analysis

**DOI:** 10.7759/cureus.64356

**Published:** 2024-07-11

**Authors:** Atitaya Apivatthakakul, Suthinee Jaruvongvanich, Sikarin Upala, Veeravich Jaruvongvanich

**Affiliations:** 1 Ophthalmology, Chiang Mai University, Chiang Mai, THA; 2 Ophthalmology, Roi et Hospital, Roi et, THA; 3 Preventive and Social Medicine, Siriraj Hospital, Bangkok, THA; 4 Gastroenterology, Queen's Medical Center, Honolulu, USA

**Keywords:** meta-analysis, vitamin d level, vitamin-d deficiency, branch retinal vein occlusion, 25 hydroxyvitamin d

## Abstract

Previous studies found seasonal variations in the incidence of retinal vascular occlusion (RVO), with more occurrence in winter. There is increasing evidence linking vitamin D deficiency and RVO. Therefore, we conducted a meta-analysis to evaluate the association between vitamin D levels and RVO. From inception to February 2024, MEDLINE and EMBASE databases were comprehensively searched. Observational studies comparing 25-hydroxyvitamin D (25(OH)D) levels between adult patients with RVO and non-RVO controls were included. We calculated pooled mean difference (MD) and pooled odds ratio (OR) with 95% confidence intervals (CI) of our data using a random-effects model and generic inverse variance method. Five studies involving 528 patients (228 patients with RVO and 300 controls were included in the meta-analysis. 25(OH)D was significantly lower in patients with RVO (pooled MD of -9.65 (95%CI -13.72 to -5.59, I2 = 92.2%). Vitamin D deficiency (serum 25(OH)D < 20) was significantly associated with RVO with the pooled OR of 14.52 (95%CI 1.72 to 122.59, I2 = 90.5). There was no difference in 25(OH)D levels between patients with central RVO and branched RVO (pooled MD of -0.94 (95%CI -3.91 to 2.03, I2 = 59.1%). In conclusion, our meta-analysis demonstrates that serum vitamin D levels were lower in patients with RVO than non-RVO controls. Clinicians could consider screening for vitamin D deficiency in patients with RVO. Further studies are warranted to determine the correlation between vitamin D levels and disease severity and the role of vitamin D supplements in these populations.

## Introduction and background

Retinal vascular occlusion [HD1] (RVO) is the second most common cause of retinal vascular disorders following diabetic retinopathy [1,2]. RVO is an obstruction of the retinal venous system, classified into two types: central RVO (CRVO) and branch RVO (BRVO), depending on the occlusion site. Three main factors, including venous stasis, hypercoagulable state, and vascular endothelial damage, have contributed to the pathogenesis of RVO [3]. A wide range of factors have been identified to increase the risk of developing RVO, for example, socioeconomic risk factors (age, gender), ophthalmic risk factors (glaucoma, ocular hypertension), and system risk factors, which also included thrombotic and atherosclerotic risk factors [4,5].

Accumulating evidence supported the seasonal variations in RVO incidence, with more occurrence during winter [6,7]. Vitamin D is a hormone with a seasonal effect with less absorption during winter, given less sunlight exposure [8]. Vitamin D is essential for skeletal health and modulates the immune/inflammation system, regulating inflammatory cytokines and oxidative stress [9]. It is known that inflammation plays a crucial role in the development of atherosclerosis. Several epidemiological studies have demonstrated an association between vitamin D deficiency and cardiovascular diseases, such as coronary artery disease, venous thrombosis, and peripheral arterial disease [10]. Given that cardiovascular diseases and RVO share common atherosclerotic risk factors and the seasonal variability in the RVO occurrence, previous publications have studied a potential link between the serum vitamin D level and RVO, which had a relatively small sample size and showed inconsistent findings [10-14]. We therefore conducted a meta-analysis to comprehensively identify all available data and summarize the available data on the association between serum vitamin D levels and RVO.

Materials and methods

This review followed the Preferred Reporting Items for Systematic Reviews and Meta-Analysis (PRISMA) guidelines [15]. This article was previously posted to the Research Square preprint server on March 30, 2024.

Search strategy

We conducted a systemic search for published articles indexed in Ovid/MEDLINE and EMBASE databases from inception to February 2024 using the search strategy that comprised terms for vitamin D and RVO as detailed in Appendices. No language restriction was applied. Reviews, case reports, and letters were excluded. References of selected retrieved articles were reviewed. A manual search for additional potentially relevant studies was also performed.

Eligibility criteria

Eligible studies must meet all of the following inclusion criteria: (1) participants must be adults (aged 18 years or older) with RVO; (2) serum 25-hydroxyvitamin (OH)D levels were measured; and (3) the study compared the 25(OH)D levels between RVO patients and controls. Two authors (AA and SJ) independently reviewed and evaluated the eligibility of the retrieved articles. The difference in the determination of eligibility of any study was resolved by consulting a third author (VJ). The corresponding authors of the included articles were contacted if additional data were required for the meta-analyses.

Data extraction and risk of bias assessment

The following data were abstracted from each study using a standardized case record form: (1) first author name, (2) year of publication, (3) study design, (4) study location, (5) number of participants, (6) participant baseline characteristics, (7) definition of controls, (8) matching factors, (9) vitamin D assessment tool, and (10) proportion of vitamin D deficiency. The quality of each study was evaluated using the Newcastle-Ottawa quality assessment scale for observational studies [16], which assessed each study in three areas, including (i) the selection of the study participants, (ii) the comparability of the groups, and (iii) the ascertainment of the outcome of interest. The score ranges from 0 to 9 stars, and a study is classified as low, moderate, and high quality with stars of 0-3, 4-6, and 7-9, respectively. Two authors (AA and SJ) independently collected data and assessed the quality of each study. Discrepancies were resolved by discussion with a third author (VJ) if required.

Statistical analysis

The outcome indicators combined in this meta-analysis mainly included binary and continuous data. When meta-analysis is performed on continuous data, the mean difference (MD) between the two groups of each study was calculated using the sample size, mean, and corresponding SDs. We analyzed the mean serum 25(OH)D between RVO cases and controls and between CRVO and BRVO patients using the pooled MD and 95%CI. If the required data was not reported directly, we conducted data processing to obtain sample size, mean, and SD using a formula for combining groups from the Cochrane Handbook for Systematic Reviews (https://handbook-5-1.cochrane.org/chapter_7/table_7_7_a_formulae_for_combining_groups.htm). For categorical outcomes, including the proportion of vitamin D deficiency in RVO cases and controls, the pooled odds ratio (OR) and 95%CI were calculated. Summary estimates with their corresponding SDs were derived using the DerSimonian and Laird method using a random effects model. The heterogeneity of effect size estimates across the studies was quantified using the Q statistic and I2 (P<0.10 was considered significant). A value of I2 of 0-25% indicates insignificant heterogeneity, 26-50% low heterogeneity, 51-75% moderate heterogeneity, and 76-100% high heterogeneity [17]. A sensitivity analysis was conducted by excluding one study from each analysis to explore each study’s influence on the overall effect size estimate. Publication bias was assessed using a funnel plot when at least 10 studies were included in the meta-analysis. Data analysis was performed using an open meta-analyst (CEBM, Brown University, Providence, Rhode Island, USA).

## Review

Results

**Figure 1:**
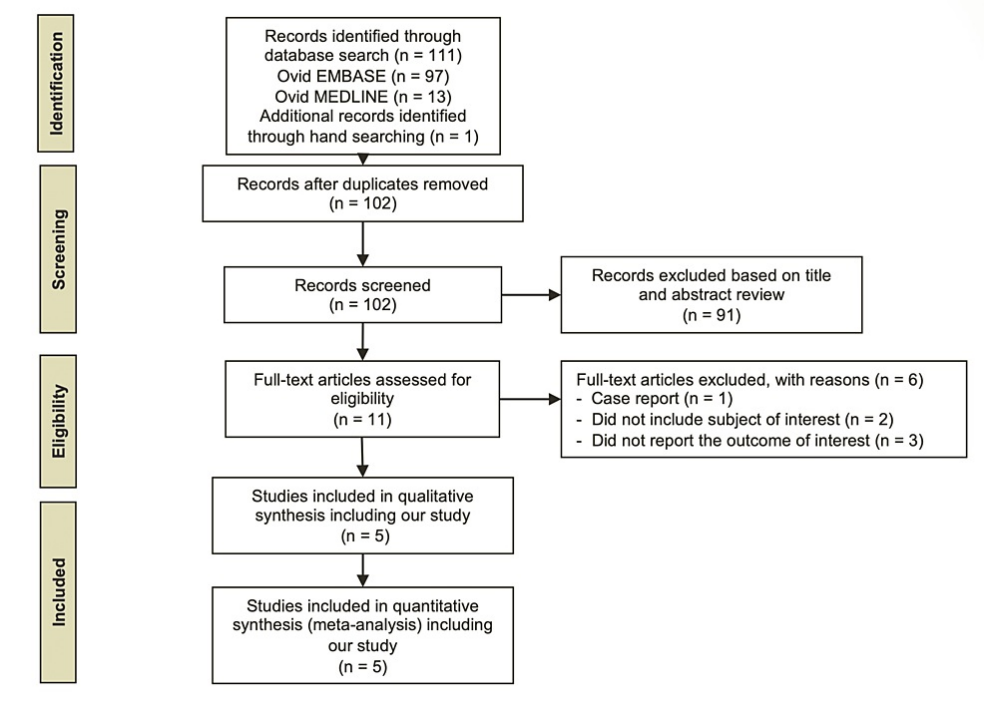
Search methodology and selection process The figure was drawn by the authors of this article.

The initial search yielded 111 potentially relevant articles (97 articles from EMBASE, 13 from MEDLINE, and one from a manual search). After excluding nine duplicated articles, 102 articles underwent title and abstract review. About 91 articles were excluded at this stage, as they did not fulfill the eligibility criteria, leaving 11 articles for full-length review. Six articles were excluded after the full-length review, for the reasons shown in Figure 1. Finally, five studies (228 cases and 300 controls) were included in the meta-analysis. All studies reported serum 25(OH)D and defined vitamin D deficiency as serum 25(OH)D lower than 20 ng/mL. The proportion of vitamin D deficiency in the included studies was high in RVO patients, ranging from 20% to 95%. The detailed characteristics of the included studies are described in Table 1. The quality score of all included studies ranged from 7 to 9, considered high quality. The quality assessment is presented in Table 2.

**Table 1:** Characteristics of the included studies BRVO: branch retinal vascular occlusion; CRVO: central retinal vascular occlusion; NA: not available; RVO: retinal vascular occlusion; SD: standard deviation

Study	Kandambeth et al. [11]	Oli and Joshi [12]	Epstein et al. [13]	Muttar and Hamiedd [14]	Bhanot et al. [18]
Year	2023	2017	2017	2022	2024
Country and setting	India, single center	India, single center	Sweden, single center	Iraq, single center	India, single center
Study design	Case-control study	Case-control study	Case-control study	Case-control study	Case-control study
Matching factors	Age	Age, gender	Age, gender, season	Age, gender	Age, gender
Total number	70	80	208	70	100
No. of cases	35	40	68	35	50
No. of controls	35	40	140	35	50
Study sample	CRVO and BRVO	CRVO and BRVO	CRVO	CRVO and BRVO	CRVO and BRVO
Controls	Subjects who met inclusion and exclusion criteria and were confirmed to be free of RVO	Attendants of patients	Randomly selected healthy subjects	Attendants of patients	Healthy subjects
Mean age (years) (mean ± SD)	60.085 ± 10.842 (cases) and 60.2 ± 10.856 (controls)	60.25 ± 9.67 (cases) and 60.73 ± 9.89 (controls)	74.1±8.7 (cases) and 73.7±7.6 (controls)	62.9% age>50 (cases) and 74.3% age>50 (controls)	62 ± 10.4 (cases) and 60 ± 9.7 (controls)
Female (%)	NA	30%	42.3%	47.1%	36%
Vitamin D measurement	Mass spectrometry	Mass spectrometry	NA	Enzyme-linked fluorescent assay	Enzyme-linked fluorescent assay
Vitamin D deficiency in RVO (%)	20%	95%	51.4%	82.9%	NA
Vitamin D deficiency in controls (%)	0%	12.5%	39.3%	22.9%	NA
Quality assessment score	7	9	9	9	9

**Table 2:** Quality assessment of the included studies *indicates allocation of score, - indicates no allocation of score

Study, Year	Selection				Comparability		Exposure				Total
	Validation of case definition	Representative of cases	Selection of controls	Definition of controls	Comparability of different samples on the basis of the design or analysis		Assessment of exposure		Same method of ascertainment for cases and controls	Same non-response rate for both groups	
	-	-	-	-	Study controls for important factor	Study controls for any additional factor	Secure record	Structured interview	-	-	
Kandambeth et al. 2023 [11]	*	*	-	*	*	-	*	-	*	*	7
Oli and Joshi 2017 [12]	*	*	*	*	*	*	*	-	*	*	9
Epstein, et al. 2017[13]	*	*	*	*	*	*	*	-	*	*	9
Muttar and Hamiedd 2022 [14]	*	*	*	*	*	*	*	-	*	*	9
Bhanot et al. 2024 [18]	*	*	*	*	*	*	*	-	*	*	9

RVO and serum 25(OH)D levels

We found that RVO patients had significantly lower serum 25(OH)D levels compared with controls with the pooled MD of -9.65 ng/mL (95%CI -13.72 to -5.59) (five studies [11-14,18]). The between-study heterogeneity was high, with an I2 of 92.2% (P-value < 0.01).

We found significantly increased odds of vitamin D deficiency among RVO patients compared to controls with the pooled OR of 14.52 (95% CI 1.72 to 122.59) (four studies [11-14]). The between-study heterogeneity was high, with an I2 of 90.5% (P-value < 0.01).

Central retinal vascular occlusion (CRVO) versus branch retinal vascular occlusion (BRVO)

**Figure 2:**
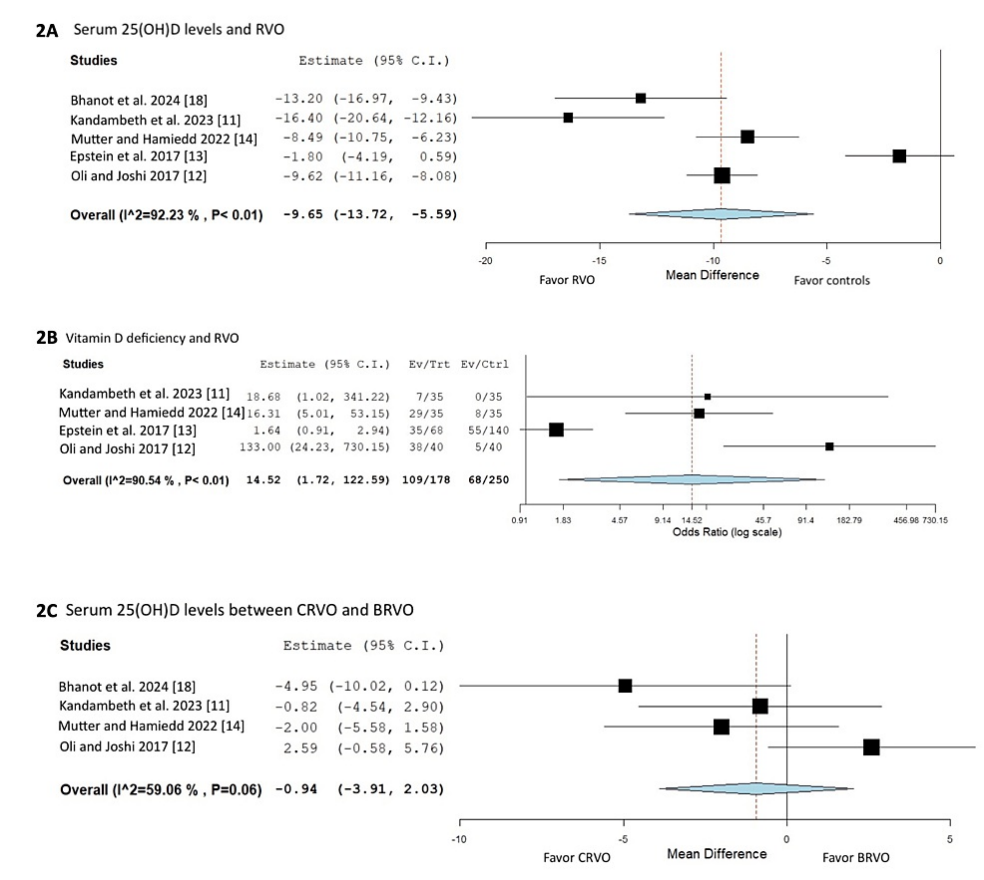
Forest plots of the included studies. (A) forest plots of mean serum 25(OH)D levels in RVO versus controls (five studies [11-14,18]), (B) forest plots of association of vitamin D deficiency and RVO (four studies [11-14]), and (C) forest plots of mean serum 25(OH)D levels in CRVO versus BRVO (four studies [11, 12, 14, 18]). BRVO: branch RVO; CI: confidence interval; CRVO: central RVO; RVO: retinal vascular occlusion

**Figure 3:**
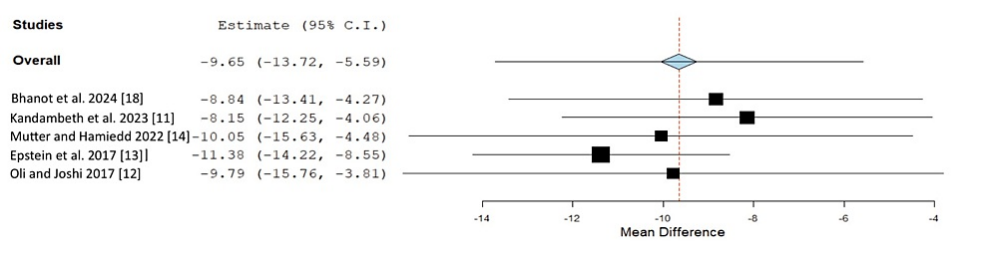
A sensitivity analysis CI: confidence interval

There was no significant difference in serum 25(OH)D levels between CRVO and BRVO patients with the pooled MD of -0.94 (95%CI -3.91 to 2.03) (four studies [11,12,14,18]). The between-study heterogeneity was moderate, with I2 of 59.1% (P-value = 0.06). The forest plots of the meta-analyses are outlined in Figure 2. In a sensitivity analysis with the exclusion of one study at a time (each MD represented the calculated MD after excluding that study), the omission of the study from Epstein et al. significantly influences the estimation of the overall effect size (Figure 3). Funnel plot analysis for publication bias assessment was not conducted because the number of included studies was not greater than 10.

Discussion

Our meta-analysis found that serum 25(OH)D levels were significantly lower in patients with RVO compared to controls, and there was no difference in serum 25(OH)D levels between patients with CRVO and BRVO subtypes.

Vitamin D has multiple roles in human health as a regulator of gene expression, immune system, inflammation, cell proliferation, differentiation, apoptosis, and angiogenesis [19]. A growing body of evidence has suggested that serum vitamin D could facilitate the development and progression of several cardiovascular disorders, for example coronary artery disease, venous thrombosis, and peripheral arterial disease [10]. A previous meta-analysis of 16 observational studies involving 47,648 individuals showed a negative relationship between vitamin D levels and the risk of venous thromboembolism, with a pooled OR of 1.74 [20]. Previous studies have also reported an association between vitamin D and multiple ocular disorders, such as uveitis, diabetic retinopathy, and macular degeneration [21,22]. A previous meta-analysis of 11 studies has shown that patients with vitamin D deficiency had 2.14-time higher odds of developing non-infectious uveitis [23]. Another meta-analysis of 14 studies demonstrated a significant association between vitamin D deficiency and diabetic retinopathy, with a pooled OR of 1.27 [24]. Previous studies have shown that the incidence of RVO was higher in winter, suggesting that low vitamin D levels may play a role [6,7]. Vitamin D action is mediated through vitamin D receptors expressed in various tissues, including vascular endothelial cells. The association between vitamin D and RVO may be explained by vitamin D’s protective role on the retinal endothelial cells from oxidative damage and inflammation, which are the pathogenic factors of RVO development [25,26]. There was a case report of CRVO with marked vitamin D deficiency in 2015 [27]. Since then, several observational studies have shown inconsistent findings [11-14,18]. Our study has demonstrated the negative association between serum vitamin D levels and the risk of RVO with a pooled OR of 14.5. with no difference between the two RVO subtypes (CRVO versus BRVO). Blood pressure tends to be higher during winter which may also contribute to higher RVO incidence [28]. Future studies need to account for the effect of blood pressure to assess the association between vitamin D and RVO incidence.

Despite the advantage of systematic review and meta-analysis technique that comprehensively summarizes all available data, this study has some limitations that may affect the validity and applicability of the pooled results. First, due to the observational nature of the included studies, the casual relationship between vitamin D and RVO could not be established. It is also subject to potential confounders. The potential confounders were minimized by the matched case-control study design. Second, the included studies have a small sample size. This could be due to the low prevalence nature of the disease. Third, there was significant in-between study heterogeneity. The sensitivity analysis using the leave-one-out method indicated that Epstein et al. [13] significantly influenced the pooled results, which could be one source of heterogeneity. The difference in a number of participants, study location and vitamin measurement methods may explain this. A previous study showed that different vitamin D measurement methods could affect the results of vitamin D levels [29]. However, the meta-analysis result remained significant despite removing the study from Epstein et al. [13].

## Conclusions

In conclusion, our study supports the association between low serum vitamin D and RVO. Vitamin D deficiency is prevalent in RVO patients. Serum vitamin D should be routinely screened in patients with RVO, along with other risk factor assessments, and vitamin D supplements should be provided if indicated. Further studies are needed to address the role of vitamin D levels on the disease severity of RVO and the impact of vitamin D supplementation on either the prevention or treatment of RVO.

## Appendices

**Table 3:** Search strategy EMBASE

1	(('vitamin d'/exp OR 'vitamin d') OR 'vitamin d*' OR ('25 hydroxyvitamin d'/exp OR '25 hydroxyvitamin d') OR ('calcitriol'/exp OR 'calcitriol') OR ('ergocalciferol'/exp OR 'ergocalciferol') OR ('colecalciferol'/exp OR 'colecalciferol') OR '25 hydroxyvitamin d' OR '1,25-dihydroxyvitamin d' OR '25(oh)d' OR '1,25(oh)(2)d' OR 'vitamin d2' OR 'vitamin d3' OR 'calcidiol' OR 'calcifediol')
2	((('retina'/exp OR 'retina') OR 'retina*')
3	(('vein'/exp OR 'vein') OR 'venous*' OR 'vessel*' OR 'vascula*' OR ('artery'/exp OR 'artery') OR 'arter*')
4	(('thrombosis'/exp OR 'thrombosis') OR ('occlusion'/exp OR 'occlusion') OR 'occlusi*'))
5	1 and 2 and 3 and 4

**Table 4:** Search strategy MEDLINE

1	Exp Vitamin D/ (69937)
2	25-hydroxyvitamin D.mp. (mp=title, book title, abstract, original title, name of substance word, subject heading word, floating sub-heading word, keyword heading word, organism supplementary concept word, protocol supplementary concept word, rare disease supplementary concept word, unique identifier, synonyms, population supplementary concept word, anatomy supplementary concept word) (17616)
3	1,25-dihydroxyvitamin D.mp. (mp=title, book title, abstract, original title, name of substance word, subject heading word, floating sub-heading word, keyword heading word, organism supplementary concept word, protocol supplementary concept word, rare disease supplementary concept word, unique identifier, synonyms, population supplementary concept word, anatomy supplementary concept word) (5116)
4	Vitamin D2.mp. (mp=title, book title, abstract, original title, name of substance word, subject heading word, floating sub-heading word, keyword heading word, organism supplementary concept word, protocol supplementary concept word, rare disease supplementary concept word, unique identifier, synonyms, population supplementary concept word, anatomy supplementary concept word) (1697)
5	Vitamin D3.mp. (mp=title, book title, abstract, original title, name of substance word, subject heading word, floating sub-heading word, keyword heading word, organism supplementary concept word, protocol supplementary concept word, rare disease supplementary concept word, unique identifier, synonyms, population supplementary concept word, anatomy supplementary concept word) (13982)
6	Ergocalciferol.mp. (mp=title, book title, abstract, original title, name of substance word, subject heading word, floating sub-heading word, keyword heading word, organism supplementary concept word, protocol supplementary concept word, rare disease supplementary concept word, unique identifier, synonyms, population supplementary concept word, anatomy supplementary concept word) (869)
7	Cholecalciferol.mp. (mp=title, book title, abstract, original title, name of substance word, subject heading word, floating sub-heading word, keyword heading word, organism supplementary concept word, protocol supplementary concept word, rare disease supplementary concept word, unique identifier, synonyms, population supplementary concept word, anatomy supplementary concept word) (10498)
8	Calcidiol.mp. (mp=title, book title, abstract, original title, name of substance word, subject heading word, floating sub-heading word, keyword heading word, organism supplementary concept word, protocol supplementary concept word, rare disease supplementary concept word, unique identifier, synonyms, population supplementary concept word, anatomy supplementary concept word) (564)
9	Calcifediol.mp. (mp=title, book title, abstract, original title, name of substance word, subject heading word, floating sub-heading word, keyword heading word, organism supplementary concept word, protocol supplementary concept word, rare disease supplementary concept word, unique identifier, synonyms, population supplementary concept word, anatomy supplementary concept word) (4854)
10	Calcitriol.mp. (mp=title, book title, abstract, original title, name of substance word, subject heading word, floating sub-heading word, keyword heading word, organism supplementary concept word, protocol supplementary concept word, rare disease supplementary concept word, unique identifier, synonyms, population supplementary concept word, anatomy supplementary concept word) (23763)
11	Vitamin D deficiency.mp. or exp vitamin D deficiency/ (39748)
12	Retina.mp. or exp retina/ (191716)
13	Retinal.mp. (231806)
14	Vein.mp. or exp veins/ (400753)
15	Venous.mp. (287456)
16	Vessel*.mp. (502727)
17	Artery.mp. or exp arteries/ (949417)
18	Artery.mp. or exp arteries/ (949417)
19	Thrombosis.mp. or exp thrombosis/ (254760)
20	Occlusio*.mp. (214732)
21	Vascula*.mp. (967803)
22	12 or 13 (296843)
23	14 or 15 or 16 or 17 or 18 or 21 (2448026)
24	19 or 20 (444082)
25	22 and 23 and 24 (14287)
26	Vitamin D*.mp. (98378)
27	1 or 2 or 3 or 4 or 5 or 6 or 7 or 8 or 9 or 10 or 11 or 26 (120259)
28	25 and 27 (13)

## References

[1] Laouri M, Chen E, Looman M, Gallagher M (2011). The burden of disease of retinal vein occlusion: Review of the literature. Eye.

[2] Song P, Xu Y, Zha M, Zhang Y, Rudan I (2019). Global epidemiology of retinal vein occlusion: A systematic review and meta-analysis of prevalence, incidence, and risk factors. J Glob Health.

[3] Zhang XT, Zhong YF, Xue YQ (2022). Clinical features of central retinal vein occlusion in young patients. Ophthalmol Ther.

[4] Kolar P (2014). Risk factors for central and branch retinal vein occlusion: A meta-analysis of published clinical data. J Ophthalmol.

[5] Bucciarelli P, Passamonti SM, Gianniello F, Artoni A, Martinelli I (2017). Thrombophilic and cardiovascular risk factors for retinal vein occlusion. Eur J Intern Med.

[6] Matsuzawa M, Sakanishi Y, Ebihara N (2020). Seasonal variation in the occurrence of retinal vein occlusion: A 4-year cross-sectional study. BMC Ophthalmol.

[7] Epstein D, Kvanta A, Lindqvist PG (2015). Seasonality and incidence of central retinal vein occlusion in Sweden: A 6-year study. Ophthalmic Epidemiol.

[8] Raymond-Lezman JR, Riskin SI (2023). Benefits and risks of sun exposure to maintain adequate vitamin D levels. Cureus.

[9] Yin K, Agrawal DK (2014). Vitamin D and inflammatory diseases. J Inflamm Res.

[10] Norman PE, Powell JT (2014). Vitamin D and cardiovascular disease. Circ Res.

[11] Kandambeth V, Nagrale P, Daigavane S (2023). Estimation of vitamin D levels in patients with retinal vein occlusions and a comparison with age-matched control groups. Cureus.

[12] Oli A, Joshi D (2017). Serum vitamin D levels in Indian patients with retinal venous occlusions. Saudi J Ophthalmol.

[13] Epstein D, Kvanta A, Lindqvist PG (2017). Vitamin D deficiency in patients with central retinal vein occlusion: A case control study. Curr Eye Res.

[14] Muttar A, Hamiedd F (2022). Asscociation between low serum vitamin D level and retinal venous occlusion at lbn-Alhaitham eye teaching hospital. AL-Qadisiyah Med J.

[15] Page MJ, McKenzie JE, Bossuyt PM (2021). The PRISMA 2020 statement: An updated guideline for reporting systematic reviews. BMJ.

[16] Stang A (2010). Critical evaluation of the Newcastle-Ottawa scale for the assessment of the quality of nonrandomized studies in meta-analyses. Eur J Epidemiol.

[17] Higgins JP, Thompson SG, Deeks JJ, Altman DG (2003). Measuring inconsistency in meta-analyses. BMJ.

[18] Bhanot R, Kumar A, Shankar S, Singh A, Ambiya V, Srujana D (2024). Serum vitamin D level alterations in retinal vascular occlusions. Photodiagnosis Photodyn Ther.

[19] Zmijewski MA (2019). Vitamin D and human health. Int J Mol Sci.

[20] Hung KC, Yang SH, Chang CY (2023). Is circulating vitamin D status associated with the risk of venous thromboembolism? A meta-analysis of observational studies. Nutrients.

[21] Chan HN, Zhang XJ, Ling XT (2022). Vitamin D and ocular diseases: A systematic review. Int J Mol Sci.

[22] Pillar S, Amer R (2022). The association between vitamin D and uveitis: A comprehensive review. Surv Ophthalmol.

[23] Rojas-Carabali W, Pineda-Sierra JS, Cifuentes-González C (2024). Vitamin D deficiency and non-infectious uveitis: A systematic review and Meta-analysis. Autoimmun Rev.

[24] Zhang J, Upala S, Sanguankeo A (2017). Relationship between vitamin D deficiency and diabetic retinopathy: A meta-analysis. Can J Ophthalmol.

[25] Song YS, Jamali N, Sorenson CM, Sheibani N (2023). Vitamin D receptor expression limits the angiogenic and inflammatory properties of retinal endothelial cells. Cells.

[26] Jamali N, Sorenson CM, Sheibani N (2018). Vitamin D and regulation of vascular cell function. Am J Physiol Heart Circ Physiol.

[27] Talcott KE, Eliott D (2016). Central retinal vein occlusion associated with severe vitamin D deficiency. Ophthalmic Surg Lasers Imaging Retina.

[28] Fares A (2013). Winter hypertension: Potential mechanisms. Int J Health Sci (Qassim).

[29] Snellman G, Melhus H, Gedeborg R, Byberg L, Berglund L, Wernroth L, Michaëlsson K (2010). Determining vitamin D status: A comparison between commercially available assays. PLoS One.

